# Next generation sequencing and *de novo* transcriptomics to study gene evolution

**DOI:** 10.1186/1746-4811-10-34

**Published:** 2014-10-20

**Authors:** Achala S Jayasena, David Secco, Kalia Bernath-Levin, Oliver Berkowitz, James Whelan, Joshua S Mylne

**Affiliations:** The University of Western Australia, School of Chemistry and Biochemistry & ARC Centre of Excellence in Plant Energy Biology, 35 Stirling Highway, Crawley Perth, 6009 Australia; La Trobe University, Department of Botany, School of Life Sciences & ARC Centre of Excellence in Plant Energy Biology, AgriBio, the Centre for AgriBioscience, 5 Ring Road, Melbourne, Bundoora Victoria 3086 Australia; The University of Western Australia, School of Plant Biology, 35 Stirling Highway, Crawley Perth, 6009 Australia

**Keywords:** *De novo* transcriptomics, Gene evolution, PawS1, Cyclic peptides

## Abstract

**Background:**

Studying gene evolution in non-model species by PCR-based approaches is limited to highly conserved genes. The plummeting cost of next generation sequencing enables the application of *de novo* transcriptomics to any species.

**Results:**

Here we describe how to apply *de novo* transcriptomics to pursue the evolution of a single gene of interest. We follow a rapidly evolving seed protein that encodes small, stable peptides. We use software that needs limited bioinformatics background and assemble four *de novo* seed transcriptomes. To demonstrate the quality of the assemblies, we confirm the predicted genes at the peptide level on one species which has over ten copies of our gene of interest. We explain strategies that favour assembly of low abundance genes, what assembly parameters help capture the maximum number of transcripts, how to develop a suite of control genes to test assembly quality and we compare several sequence depths to optimise cost and data volume.

**Conclusions:**

*De novo* transcriptomics is an effective approach for studying gene evolution in species for which genome support is lacking.

## Background

### *De novo*transcriptomics

Next generation sequencing (NGS) technology and the accompanying drop in the per-base cost of the sequence data has enabled many new approaches in molecular biology such as whole genome sequencing [1], transcriptomics [2], metagenomics [3], epigenomics [4], proteomic applications such as ProteinSeq [5], and single-cell sequencing [6]. Before NGS, transcriptomics was largely limited to model species using oligo arrays hybridised to transcript libraries. NGS has allowed for transcriptomic studies leading to gene discovery [2, 7], SNP detection [8], simple sequence repeat discovery [9], and gene pathway description [10]. The challenge with non-model transcriptomes is a bioinformatic one, they need to be assembled without any reference genomes *i.e. de novo*. One approach to avoid *de novo* assembly is to assemble the transcriptomes of a non-model species using a partial reference-based strategy with sequences from a closely related model species [11].

*De novo* transcriptome assembly will continue to become less challenging as the length of NGS reads increases [12] and as software develops better algorithms for unsupported assemblies. A wealth of open source and LINUX-based software developed for transcriptome assembly exist including Trinity [13], SOAPdenovo (http://soap.genomics.org.cn/soapdenovo.html), Velvet [14], CAP3 [15], and TGICL [16]. Commercial software for the mainstream operating systems also exists with examples including CLC Genomics Workbench (CLC bio), SeqMan Ngen (DNASTAR), and gsAssembler (Life Technologies).

### The *PawS1*gene

**Figure 1:**
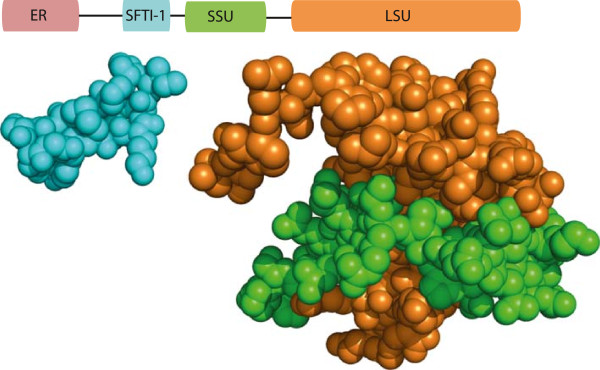
**Structure of the sunflower preproalbumin PawS1 and its mature proteins.** PawS1 is matured into two proteins [17] represented here as sphere-formatted structural models; a small peptide called SFTI-1 (cyan, PDB 1sfi [20]) and a heterodimeric seed storage albumin consisting of a small subunit (SSU, green) and a large subunit (LSU, orange), here represented by a *Brassica napus* napin structure (PDB BnIb [21]).

*De novo* transcriptomics is being used to study the evolution of the *PawS1* (Preproalbumin with SFTI-1) genes, which were first discovered in sunflower (*Helianthus annuus*) [17]. Sunflower PawS1 (151-residue protein) and a close relative PawS2 (137-residue protein) have an unusual dual biosynthesis in that each are matured into two very different proteins; a 10.5 kDa napin-type seed storage albumin and an ‘extra’ 1.5 kDa cyclic peptide that is buried in the proalbumin sequence (Figure 1) [17]. To study *PawS1* evolution, genes have been amplified using PCR from related species with primers designed against genomic DNA sequence flanking the *PawS1* and *PawS2* genes. This approach identified *PawS1* genes from Asteraceae members related to sunflower and defined a new class of seed peptide termed “PawS-Derived Peptides” (PDPs) that is at least 18 million years old [18]. Seed storage proteins are already known to evolve rapidly [19], but the PDP region in *PawS1* genes evolved faster than the adjacent albumin [18]. The PCR-based amplification strategy also discovered some *PawS*-*like* (*PawL1*) genes that share many features with *PawS1*, but the PDP region does not produce a stable peptide and is proposed to be an ancestral form of *PawS1* [18].

The heterologous PCR approach is limited in that it cannot amplify *PawS1* genes from Asteraceae species that are more distantly related to sunflower. Very little sequence information is available for the species in which *PawS1* genes are present. The first draft of the sunflower genome is pending and has been challenging due to its large size (3,600 Mbp) and high (81%) content of transposable elements [22]. In Elliott *et al*. [18] *de novo* transcriptomes for sunflower and mountain arnica (also known as leopard’s bane, wolf’s bane, and mountain tobacco; *Arnica montana*) indicated NGS was a viable approach to study the evolution of *PawS1*. We have subsequently assembled *de novo* transcriptomes for dozens of seed RNA samples and have found by cloning the putative genes and finding supporting peptide evidence by mass spectrometry that NGS-based study of single genes is a viable approach. Among the species analysed, *Zinnia haageana* (also known as Persian carpet, Mexican Zinnia, dwarf Zinnia) was particularly challenging due to presence of a large number of closely-related *PawS1* genes.

### Drawbacks of NGS-based gene discovery

NGS is not the best approach for all studies of gene evolution. If the gene sequence of interest is highly conserved, a PCR approach remains the most cost-effective. Quality control of NGS data is critical due to the potential for sequence or assembly errors. Cloning large number of genes, even with primers designed from the assembled sequences can be laborious, especially if the assembled transcript does not contain a complete open reading frame (ORF). RNA-seq is of course dependent on the gene being transcribed so it will not detect gene evolution that triggers a loss in gene expression. Finally for labs to adopt this approach there are the obvious bioinformatic barriers, the most obvious being the handling and processing of millions of reads which might require new computational skills or software in addition to new hardware to store the data - formidable considering the target gene constitutes only a very small proportion of the reads gathered.

### Benefits of an NGS-based approach to studying gene evolution

Despite these drawbacks, applying NGS has its benefits; (i) The type of sequencing can be selected based on the objective. To assemble transcriptomes from higher organisms, high data volumes are required, so they are usually sequenced using either Illumina or SOLiD technologies (reviewed in [23]). The 454 platform creates longer reads, but the error rate is relatively high (reviewed in [24]). (ii) Software, such as CLC Genomics Workbench (CLC bio) is designed for users without extensive bioinformatics training. This software had been used by different groups to assemble *de novo* transcriptomes in non-model plants [25, 26]. (iii) NGS-based methods can provide the full ORF and this is preferable for situations where PCR primers may only be designed within ORFs and must be followed by 3’ and 5’ RACE; (iv) NGS can discover transcripts in any species for which RNA can be purified and so permits studying fast-evolving genes; (v) A suite of control genes ranging in expression can indicate whether the absence of a specific transcript is due to either a poor assembly or poor data for assembly; (vi) RNA-seq by NGS provides the expression level of transcripts [27], and (vii) Unlike microarray based transcriptomic approaches, RNA-seq by NGS can also find splice variants if present [28].

Although we have an interest in the evolution of the *PawS1* gene [18], *PawS1* also serves as a good test case for the application of *de novo* transcriptomics to track gene evolution. PawS1 is a precursor for albumin and seed storage genes in general have been shown to evolve rapidly [19]. PawS1 also encodes a peptide whose mass can be detected readily using mass spectrometry, proving proteinacous evidence for assembled transcripts. Finally, the *PawS1* gene is found in sunflower, for which no genome is currently available and is in a plant family studied little with genomic approaches. Most species we have tested contain only one or two *PawS1* genes, but we found that *Z. haageana* has many and this offered an ideal dataset to test the ability to separate related genes and test the parameters for gene discovery.

Here, using four Illumina RNA-seq datasets (including the challenging *Z. haageana*) the bioinformatic strategies adopted to discover *PawS1* transcripts are outlined, the selection of a suite of control genes to evaluate the quality of seed-RNA assemblies are detailed to help explain why a *PawS1* gene might not be present in an assembled transcriptome. These strategies can be used to develop an NGS approach to studying the evolution of any gene in model and non-model species.

## Results and discussion

### *De novo*transcriptomics approach for *PawS1*genes

To demonstrate the use of NGS to study gene evolution, two recently published and publicly available datasets originating from dry-seed mRNA of *H. annuus* (SAMN02569067) [18] and *A. montana* (SAMN02569068) [18] were used. Additionally new sequence data from dry-seed mRNA of *Z. haageana* (SAMN02933922) and *Heliopsis helianthodes* (commonly known as smooth oxeye or false sunflower) (SAMN02933923) were also obtained. All sequence data were paired-end reads. The reads were assembled using CLC Genomics Workbench 6.5.1 (CLC bio).

### Quality control of the reads

Although CLC Genomics can clean reads during import, the open source FASTX toolkit (http://hannonlab.cshl.edu/fastx_toolkit/) was used for read trimming and filtering as it offers more flexibility to define quality thresholds.

Table 1 shows the number of clean reads obtained after trimming and filtering. For *Z. haageana* and *H. helianthoides* datasets, trimming parameters were maintained as quality threshold of the read ends (t) at 30 and the minimum length after trimming (l) at 50. A quality threshold (t) of 30 means the probability of incorrect base call is 1 in 1,000 bases or the base call accuracy is 99.9%. A minimum length after trimming (l) of 50 means reads shorter than 50 bp after trimming will be discarded.

**Table 1:** **Assembly statistics**

Species	Read length	Raw reads	Clean reads	Assembler	N50	Contig count
*H. annuus*	101	2 x 21,404,702	40,742,686	CLC (ws60,paired)	482	59,530
*A. montana*	101	2 x 14,458,043	27,516,042	CLC (ws60,paired)	485	45,194
*Z. haageana*	101	2 x 38,382,090	64,649,107	CLC (autows,non-paired)	308	205,324
*Z. haageana*	101	2 x 38,382,090	64,649,107	CLC (ws60,paired)	435	80,460
*Z. haageana*	101	2 x 38,382,090	72,756,408	CLC (ws60,paired)	629	40,764
*H. helianthoides*	101	2 x 109,627,594	169,128,716	CLC (autows,non-paired)	305	443,800
*H. helianthoides*	101	2 x 109,627,594	169,128,716	CLC (ws60,paired)	497	151,272
*H. helianthoides*	101	2 x 109,627,594	200,130,791	CLC (ws60,paired)	496	162,563

Clean reads were assembled using two methods; automatic word size (autows, 23), non-paired and word size 60 (ws60), paired method. Number of clean reads when quality filtering was done to achieve a quality threshold (q) of 30 and 22 are shown for *Z. haageana* and *H. helianthoides* datasets. N50 refers to the contig length where 50% of the assembly is represented by contigs of this size or longer.

Filtering parameters for these two datasets were set as q30, p90. A quality threshold (q) of 30 means the base call accuracy is 99.9%. The minimum percentage of bases that must match (p) the specified quality threshold (in this case q30) is 90. For example, quality filtering of the *H. helianthoides* dataset to achieve a quality threshold of 22 (q22, p90) removed only 8% reads compared to 22% when q30, p90 is used (Table 1). A quality threshold of 20 (q20) equals to a base call accuracy of 99%. Similarly 15% of raw reads were removed when quality filtering parameters were set at q30, p90 in the *Z. haageana* dataset. Less stringent filtering parameters (*i.e*. q22, p90) removed only 5% of the raw reads.

Clean reads from both *H. helianthoides* and *Z. haageana* under the two different quality filtering parameters were assembled with the word size 60, paired method described below. The assemblies were queried for PawS1 using tBLASTn. Two different quality filtering parameters did not affect the number of *PawS*/*PawL* transcripts found in both species.

However, it was observed that quality filtering with less stringent parameters followed by assembly helped obtain longer *PawS*/*PawL* transcripts, and sometimes identified novel transcripts in some datasets.

### Performing the assembly

**Figure 2:**
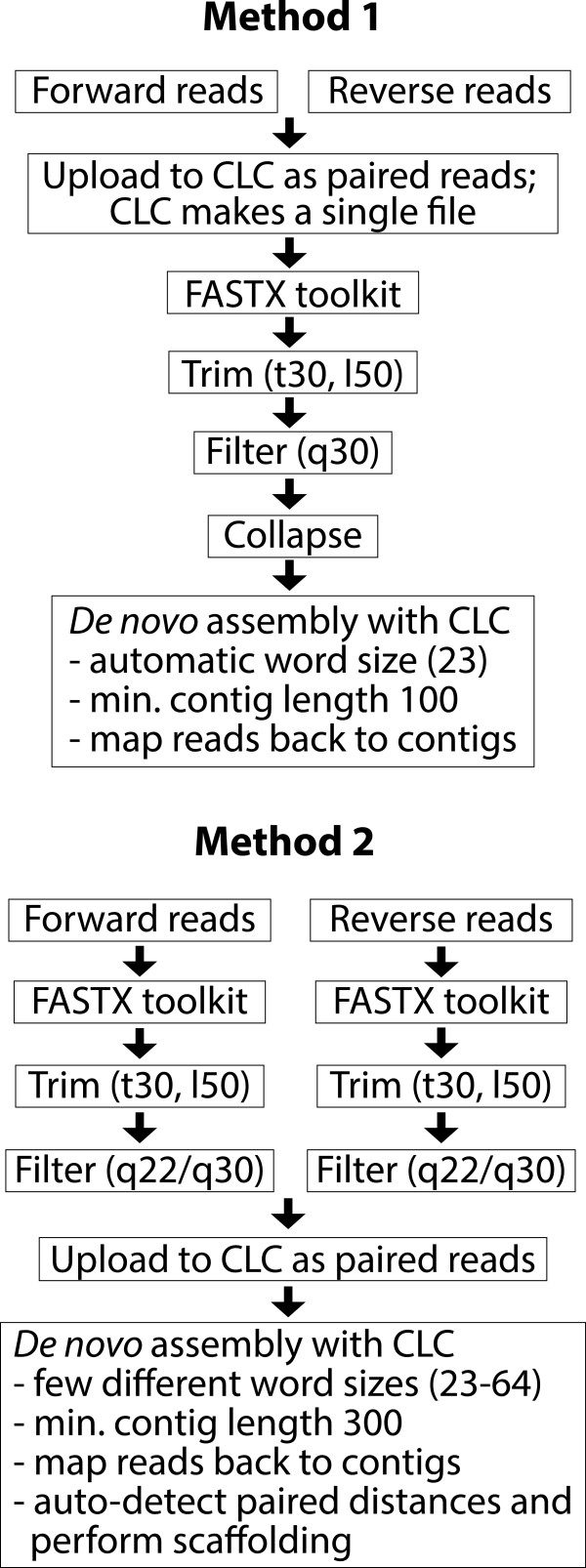
**Flow diagram showing the two different assembly methods used.** In the first method raw reads were combined before trimming, filtering, and collapsing. Then they were assembled using CLC Genomics using the automatic word size (23) set by the program indicating the minimum contig length as 100. In the second method forward and reverse reads were trimmed and filtered individually. High or low quality filtering parameters were tried. Trimmed and filtered reads were assembled using CLC Genomics. Several different word sizes were tried and the minimum contig length was set to 300. Paired distances were automatically detected by the program and scaffolding was performed.

To find the optimum CLC parameters to identify the maximum number of *PawS*/*PawL* transcripts, we assembled *H. helianthoides* and *Z. haageana* data using two different methods (Figure 2). Initially all forward and reverse paired-end reads were combined and assembled under CLC default settings where the word size (*k*-*mer*) is automatically set by the program, indicating the minimum contig length as 100. The word size automatically set by the program was 23 for both datasets. In this case paired information was not used for assembly. In the second method, paired information was used and a range of word sizes was tested as well as setting the minimum contig length to 300, instead of the default 200. The number of *PawS*/*PawL* transcripts found in assemblies with different word sizes varied greatly and among the word sizes tested, 60 gave the maximum number of transcripts from all four RNA-seq datasets.

### Development of a suite of core transcripts

Traditional quality metrics such as N50 (N50 refers to the contig length where 50% of the assembly is represented by contigs of this size or longer) and contig count are often used to represent the quality of *de novo* assemblies, but such figures can be mis-leading [29, 30]. Parra *et al*. [30] have described a more reliable method to assess the quality of *de novo* assembled genomes. The most important outcome from a genome or a transcriptome is their catalogue of genes. So Parra *et al*. [30] used a set of core genes which should be present in all eukaryotic genomes as controls and analysed how completely these core genes are assembled in eukaryotic genomes assembled using different methods.

We also used a similar approach to assess the quality of our *de novo* transcriptome assemblies. The CLC *de novo* assembly tool has the option to map reads back to contigs permitting an evaluation of abundance. This permits selection of transcripts with a range of expression levels from high to low.

We assembled the sunflower transcriptome using a word size of 60 with paired-end reads and mapped the reads back to contigs and selected subsets of contigs to represent high, medium, and low levels. To identify the transcripts represented by these contigs we used the BLAST at NCBI tool menu in CLC. Examining these BLAST hits identified a suite of six core genes (Table 2) expressed in seeds. Dozens of Asteraceae seed *de novo* transcriptomes were queried for these core genes to make sure they were conserved in this plant family. The six include transcripts expressed at different levels and included long and short transcripts. Ideal core transcripts for this purpose would be highly conserved single-copy genes. However, without genome support, it was challenging to find such transcripts for Asteraceae members.

**Table 2:** **Details of the core genes used to assess the quality of** *de novo* **transcriptome assemblies**

Gene name	GenBank ID	Length (amino acid)	Average coverage
*Late embryogenesis abundant* (*LEA*)	X59700.1	104	174,360
*Oleosin* (*OLE*)	X78679.1	183	9,894
*Aspartic proteinase* (*AP*)	AB025359.2	509	1,561
*Pathogenesis related* (*PR*)	AB091075.1	158	503
*Cysteine protease*-*1* (*CP*-*1*)	AB109186.1	461	126
*Serine*/*threonine protein kinase* (*PK*)	AB090881.1	439	50

The level of transcription in sunflower ranges from high (*LEA*) to low (*PK*). The GenBank ID for the amino acid sequence used to tBLASTn each *de novo* transcriptome is shown. The average coverage in a word size 60, paired method transcriptome provides an indication of their relative abundance. This control set was found to be appropriate for the Asteraceae. For reference, the most closely related sequences in *Arabidopsis thaliana* are *LEA4*-*5* (At5g06760), an *OLEOSIN* family member (At3g01570), *APA1* (At1g11910), *MLP423* (At1g24020), *RD21B* (At5g43060), and *Protein kinase* (*PK*) super family member (At5g15080).

The first core transcript we selected was a highly expressed gene belonging to the *LATE EMBRYOGENESIS ABUNDANT* (*LEA*) family. This *LEA* family transcript was found to be ubiquitous in the sunflower mature (dry) seed *de novo* transcriptome. LEA protein is generally formed during the latter part of seed development and was first described in cotton seeds [31], but later found in seeds of many other land plants, also in vegetative tissues. LEA proteins are considered to be involved in plant stress and dehydration tolerance (reviewed in [32]).

The second core transcript was an *OLEOSIN* (*OLE*) gene family member. Oil in seeds is normally stored in oil bodies composed of oleosin proteins that control their structure and lipid accumulation [33]. The majority of Asteraceae are oilseeds and we found this *OLE* to be ubiquitous and highly expressed.

The third core transcript was an *ASPARTIC PROTEINASE* (*AP*). *AP*s are widely distributed in the plant kingdom and found in many plant tissues including seeds [34]. Plant aspartic proteinases are thought to be involved in protein processing, protein degradation, senescence, stress responses, and sexual reproduction (reviewed in [35]). This particular *AP* was moderately expressed in sunflower.

The fourth core transcript was a *PATHOGENESIS RELATED* (*PR*) gene. PRs in plants are normally involved in defence signalling. PR proteins are induced by plant hormones such as jasmonic acid, ethylene, and salicylic acid (reviewed in [36]). *PR* has low expression in sunflower and is the most similar in expression to *PawS1*.

The fifth core transcript we selected was a *CYSTEINE PROTEASE*-*1* (*CP*-*1*). The closest member from *Arabidopsis* to this *CP*-*1 is RD21B* which is a plant papain-like cysteine protease. Plant papain-like cysteine proteases are involved in plant defence, development, and senescence [37]. *CP*-*1* is a low abundance transcript in sunflower.

The sixth core transcript was a *SERINE*/*THREONINE PROTEIN KINASE* (*PK*). PKs are widely distributed in the plant kingdom and are found to be involved in many signalling cascades such as plant hormone signalling, defence responses or tolerance to stresses [38]. This *PK* had the lowest abundance among the six core transcripts.

Of the core transcripts *CP*-*1* and *PK* were typically expressed at a lower level than our target gene *PawS1* (Table 3) and therefore were the most valuable indicators for the quality of assembly. Presence of *CP*-*1* and *PK* contigs suggested a high likelihood that transcripts with similar expression levels such as *PawS1*/*PawL1*, would be included in our assemblies. This was especially important for species where assemblies lacked *PawS1*/*PawL1* genes.

**Table 3:** ***De novo*** **transcriptome assembly quality statistics**

Gene	***H. annuus***	***A. montana***	***Z. haageana***	***Z. haageana***	***H. helianthoides***	***H. helianthoides***
	ws60, paired	ws60, paired	autows, non-paired	ws60, paired	autows, non-paired	ws60, paired
*LEA*	90%	80%	97%	79%	53%	73%
	165,041	23	25,957	5,584	162,273	176,283
*OLE*	100%	100%	57%	95%	100%	45%
	14,242	23,511	108,400	12,436	114,811	145,334
*AP*	78%	100%	63%	89%	97%	97%
	1,666	405	237	43	5,704	5,704
*PR*	100%	100%	89%	100%	79%	100%
	4,922	6,183	6	13	44,849	36,182
*CP*-*1*	51%	72%	100%	98%	100%	94%
	122	44	70	66	363	355
*PK*	73%	63%	83%	97%	75%	99%
	63	48	59	40	175	229
*PawS1a*	100%	-	100%	100%	70%	70%
	532		3,862	3,362	1,827	1,827
*PawS1b*	84%	-	79%	100%	32%	40%
	221		809	927	88	86
*PawS1c*	-	-	29%	58%	32%	41%
			15	58	798	782
*PawS1d*	-	-	70%	41%	-	-
			19,930	7,916		
*PawS1e*	-	-	33%	36%	-	-
			2,236	7,394		
*PawS1f*	-	-	33%	42%	-	-
			2,142	2,518		
*PawS1g*	-	-	44%	-	-	-
			7,071			
*PawS1h*	-	-	-	37%	-	-
				129		
*PawS1i*	-	-	30%	-	-	-
			550			
*PawS1j*	-	-	-	40%	-	-
				30		
*PawS1k*	-	-	22%	52%	-	-
			4	8690		
*PawL1a*	98%	100%	100%	-	100%	100%
	25	400	4		4,711	4,711
*PawL1b*	-	-	40%	87%	-	-
			4,125	4,736		

All four species were assembled with word size (ws) 60, paired method. In addition, *Z. haageana* and *H. helianthoides* transcriptomes were assembled with the automatic word size (autows, 23), non-paired method. The first line shows the percentage coverage of the identified transcripts when compared to the expected full length protein query. The second line shows the average coverage by mapping trimmed, filtered reads on identified full/partial transcripts. Average coverage means sum of the bases of the aligned part of all the reads divided by the length of the reference sequence.

Using tBLASTn we identified the corresponding core transcripts in each of our *de novo* assembled seed transcriptomes and calculated the percentage of ORF coverage of each core transcript compared to the query. PawS1 is a shorter protein of around 151 amino acids, but all the core transcripts except *LEA* encode proteins that are larger than PawS1 (Table 2). Mapping raw reads to each core transcript (as well as *PawS*/*PawL* transcripts if present) provided an indication of the relative abundance.

Caution must be used with core transcripts and it is preferable to rely on a suite rather than one or two transcripts. As an example in *Z. haageana* only 57% of *OLEOSIN* is assembled with the automatic word size, non-paired method despite average coverage being very high at 108,400 (Table 3). With word size of 60, paired method, 95% of the transcript is assembled, but the average coverage drops to 12,436 (Table 3). Subtly different gene duplicates and even alleles can confound the assemblies. We selected the transcript with the best matching score from the tBLASTn output to the GenBank ID in Table 2. Many of the control genes are members of multigene families (*e.g*. there are four *OLEOSIN* genes in *Arabidopsis*) which might explain the disparity of results we observed.

### Discovering novel *PawS*/*PawL*transcripts

We used sunflower PawS1 protein sequence as a query to search all assemblies for novel *PawS*/*PawL* transcripts with the tBLASTn procedure. Precursors of napin-type seed storage albumins typically contain an endoplasmic reticulum (ER) signal and a spacer region which are discarded during the protein maturation process, followed by a polypeptide chain. The polypeptide chain is composed of two subunits separated by a spacer region (Figure 1). The small subunit typically has two conserved cysteines and the large subunit six conserved cysteines (reviewed in [39]). Typically in PawS1 proteins, a short stretch of amino acids with two conserved Cys residues are buried between the spacer region and the small subunit followed by a four amino acid tail (in most cases these four amino acids are Gly-Leu-Asp-Asn) [17, 18]. Furthermore, most of these buried peptides (PDPs) starts with a Gly residue and ends with Asp [18]. PawL1 structure is very similar PawS1 except that the buried amino acid sequence does not have the two conserved Cys residues and does not produce a stable peptide [18]. We looked for these characteristic amino acid arrangements when defining novel *PawS*/*PawL* transcripts from our assemblies.

Elliott *et al*. [18] found *PawS1*, *PawS2*, and *PawL1* in sunflower and *PawL1* in *A. montana* seed *de novo* transcriptomes assembled using Trinity. The same transcripts were detected using our assembly, but no additional *PawS*/*PawL* by assembling the two datasets with CLC using word size 60, paired method.

For *H. helianthoides* the same three *PawS1* genes were identified as in Elliott *et al*. [18] amplified using PCR. *H. helianthoides PawS1a* and *PawS1b* gene sequences are very similar to each other and differ only by two nucleotides causing protein sequences to differ in two amino acids. In addition to those three *PawS1* genes, we identified a previously unknown *PawL1* transcript. These four transcripts were present in both the automatic word size, non-paired assembly as well as in the word size 60, paired assembly. This is a good indication that CLC assemblies are reliable in terms of identifying novel *PawS*/*PawL* transcripts.

**Figure 3:**
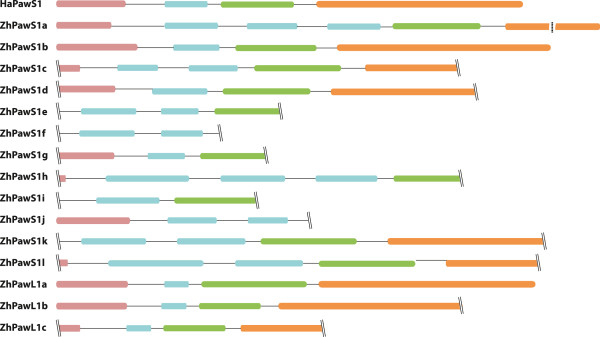
**Putative** ***Z. haageana*** **PawS1 and PawL1 proteins identified by** ***de novo*** **transcriptomics.** Colours indicate ER signal (pink), spacer regions (black), the peptide-coding regions (aqua), albumin small subunit (green), albumin large subunit (orange). Predictions of regions are based on comparison to sunflower PawS1. Partial transcripts are indicated by two lines. For the purpose of this figure, only part of the predicted large albumin subunit in the greatly expanded ZhPawS1a was displayed (dashed line).

In the *Z. haageana* transcriptome, 15 putative *PawS1* and *PawL1* transcripts were identified (Figure 3). However, only twelve transcripts were present in the automatic word size, non-paired assembly (Table 3). The word size 60, paired assembly identified ten transcripts (Table 3). *ZhPawS1g*, *ZhPawS1i*, and *ZhPawL1a* identified from the initial assembly were not found from the word size 60 assembly. Meantime *ZhPawS1h* and *ZhPawS1j* were only detected in the word size 60 assembly. *ZhPawS1l* could be detected when a word size of 62 was used. Similarly *ZhPawLc* was found only in word size 45 assembly.

Word size had a considerable impact on the outcome from different assemblies. Haznedaroglu *et al*. [40] also reported that each individual *k*-*mer* value assembly is missing certain biological information. Assembling with several different word sizes will ensure the capture of a maximum number of transcripts. A dataset of around 50 million reads could be assembled within 2–3 hours using CLC Genomics running on a high-speed desktop computer, meaning that several word sizes could be tested throughout a day before selecting the best assembly for a given dataset. In terms of other quality parameters of *de novo* transcriptomes such as contig length, hybrid assemblies, redundancy reduction, and error tolerance, it was shown that CLC Genomics and TGICL pipeline gave better assemblies when different software available for assembling short reads generated by NGS were compared [26].

When comparing the two methods used to assemble *Z. haageana* and *H. helianthoides* datasets we observed that automatic word size (23), non-paired assemblies where minimum contig length was set to 100 created a larger number of contigs when compared to the word size 60, paired assemblies with a minimum contig length of 300 (Table 1). This might be because assemblies with the first method keep all transcripts longer than 100 bp, while the latter removes transcripts shorter than 300 bp. Also the word size (23 vs 60) makes a difference in the number of contigs created. The number of contigs is an important consideration in a *de novo* transcriptome, but when the focus is on a single gene, it is worth retaining biological information to avoid possible loss of information. It is worth noting that our target gene is around 400–600 bp long. We can identify the PDP region which is typically around 14 amino acids, followed by its characteristic tail even in partially assembled shorter transcripts. So it is beneficial to set the minimum contig length to a lower value and also to try few different word sizes as mentioned above to minimize the loss of biological information for this type of study.

The *H. helianthoides* mRNA was sequenced in greater depth (>200 million raw reads) than the other three species (Table 1). Despite the greater depth, the same genes were identified as previously described by Elliott *et al*. [18] in *H. helianthoides* as well as in sunflower and *A. montana* where sequencing depth was moderate (Table 1). In addition to these two paired-end assemblies described here, several other Asteraceae seed *de novo* transcriptomes have been assembled with single-end reads (unpublished). Moderate or even low depth, single-end read assemblies were not inferior to paired-end assemblies in terms of discovering novel *PawS*/*PawL* transcripts as well as assembling the suite of core transcripts selected.

### Cloning and LC-MS

**Figure 4:**
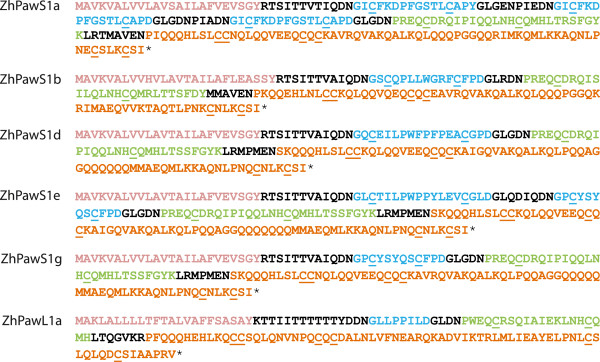
**Sequences encoded by** ***Z. haageana PawS1*** **and** ***PawL1*** **genes confirmed by PCR.** Colour scheme as Figure 2. Region delimitation is inferred by comparison to sunflower PawS1.

*Z. haageana PawS*/*PawL* genes have not been described in previous studies. To confirm the transcripts identified from the *Z. haageana* seed *de novo* transcriptome are not a result of mis-assembly, PCR was used to clone the full ORF of five of these sequences (*PawS1a*, *b*, *d*, *e*, *g*) and one *PawL1* sequence (*PawL1a*) from seed genomic DNA (Figure 4). Sequence similarity at the nucleotide level between the cloned products and their corresponding assembled transcript was 100% for *PawS1b*, *PawS1e*, *PawS1g*, *PawL1a*, 98% for *PawS1a*, and 91% for *PawS1d*. Partial, overlapping transcripts were identified for *PawS1d* from assemblies with two different word sizes. The full length sequence could be manually reconstructed by combining these partial, overlapping sequences. Primers for *PawS1d* were designed based on this contig. This might explain the relatively higher error rate observed between the cloned *PawS1d* sequence and the transcript from the assembly.

**Figure 5:**
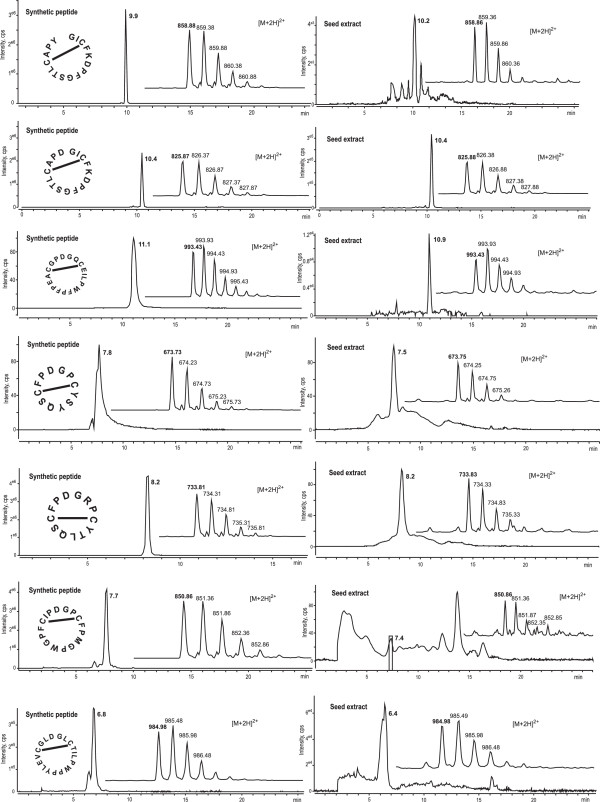
***In planta*** **confirmation of PDPs in peptide extracts of** ***Zinnia haageana*** **dry seeds.** Left side panel is the Extracted Ion Chromatograms (XICs) and average mass spectrums (inset) of the synthetic peptides showing the [M + 2H]^2+^ ions. Average retention time for each peptide is shown in minutes next to the XIC. Right side panels are XICs and mass spectrums of corresponding native peptides identified from the *Z. haageana* seed extract run under the same conditions.

Furthermore, LC-MS was used to provide evidence for these newly discovered *Z. haageana* PDPs at the protein level. The *Z. haageana PawS1* transcripts appear to give rise to seventeen unique PDPs (certain PDPs were repeated in the same gene or in different genes). By extracting peptides from the same seeds and analysing the resulting peptide LC-MS profile we found matching masses for eleven of the seventeen peptides predicted from *PawS1* sequences (Table 4). Synthetic peptides for seven of these peptides were compared to native extracts confirming their existence in LC-MS by displaying the same mass and retention time (Figure 5).

**Table 4:** ***Zinnia haageana*** **PDPs matching masses observed in the LC**-**MS profile**

Peptide sequence	Predicted mass (Da)	Observed mass (Da)	LC matches synthetic
GICFKDPFGSTLCAPY	858.88	858.86	Yes
cyclo-GICFKDPFGSTLCAPD	825.86	825.88	Yes
cyclo-GQCEILPWFPFPEACGPD	993.43	993.43	Yes
cyclo-GPCYSYQSCFPD	673.75	673.75	Yes
cyclo-GRPCYTLQSCFPD	733.81	733.83	Yes
cyclo-GLCTILPWPPYLEVCGLD	984.98	984.98	Yes
cyclo-GPCFPMGPWGPFCIPD	850.86	850.86	Yes
cyclo-GRGCFGFPPICFPD	746.83	746.83	ND
cyclo-SAACSHLPPGLREMCAAWSFD	1114.99	1114.97	ND
cyclo-SAACSHLQPVLREMCVARFD	1107.02	1107.01	ND
cyclo-GAACSHIEPGLREMCAAWSFFD	1189.51	1189.48	ND

The predicted mass is for the doubly charged [M^+2^] ion and assumes the Cys residues form a disulfide bond (oxidised). For cyclic peptides the monoisotopic mass is reduced by 18 Da. The observed masses from LC-MS experiments are calculated from the doubly charged [M^+2^] ions. Seven synthetic peptides were ordered, which allowed additional confirmation of the retention time of the mass during liquid chromatography (LC). The retention times for some masses were not confirmed using synthetic peptides (ND).

## Conclusions

Many groups have used *de novo* transcriptomic approaches for gene discovery in non-model species. Examples include identification of: genes encoding metabolites with biotechnological value [7], pigment pathway genes in spiders [41], phosphate starvation-responsive genes in wheat [2], genes differentially expressed during limb regeneration in salamanders [42], and genes responsible for agronomic traits in pennycress [25]. Comprehensive RNA-seq development resources are becoming available and include that of Wit *et al.* [43], which extends to population genomics. Here we employ a highly focused application for *de novo* transcriptomics to study gene evolution. With the cost of RNA-seq lowering, we believe it is an effective way to study even a single gene.

With the *H. annuus*, *A. montana*, and *H. helinathoides* data we showed that *PawS1* genes previously identified using the traditional PCR approach could be readily detected. Additionally when a *de novo* transcriptomics approach was used a novel *PawL1* transcript could be captured from *H. annuus* [18] and in this work, a *PawL1* transcript from *H. helianthoides*. Seed mRNA for *Zinnia haageana* was also sequenced, and this species displayed extensive duplication of *PawS1* which could make assemblies challenging. This same complexity, however allowed us to demonstrate how parameters such as the stringency of quality filtering, word size, assembly method, and the depth of sequencing affected transcript identification. A method to assess the quality of an assembly using core transcripts was also outlined. A commercial software package was sufficient and it could parse closely related genes into separate contigs.

In summary, we outline a comprehensive protocol with commercial software ideal for users with minimal bioinformatics experience and explain how to take seed total RNA through to unsupported assemblies. We found that to improve the likelihood of finding a specific gene, it was beneficial to try several different word sizes during assembly. We also detail how we developed our own set of control genes that varied in expression level among Asteraceae seeds and how we used them to assess quality. Thus the approaches described here are adaptable for laboratories that traditionally do not have expertise with NGS large datasets. Our data illustrate how *de novo* transcriptomics is a viable approach in non-model species for tracking the evolutionary history of a target gene.

## Methods

### RNA extractions

Total RNA was extracted from mature seeds of *Z. haageana* and *H. helianthodes* using the mini-hot phenol method [44], which is based on a method by Botella *et al*. [45]. RNA was DNAse treated followed by further purification using the NucleoSpin RNA Clean-up kit (Macherey-Nagel). The quality of the RNA was tested by measuring the A260/280 and A260/230 ratios using a NanoDrop Spectrophotometer and visualizing on a 1% agarose gel. When visualising total RNA extracted from dry seeds on a gel, note that the rRNA bands are not as prominent as they are for leaf total RNA, but if the RNA is intact it will similarly migrate with discrete banding. Following the above extraction and clean up procedure, some seed RNA samples will still contain pigments, but we do not find these to noticeably inhibit subsequent RNA-seq or reverse transcription.

### Library construction

Sequencing libraries were generated using the TruSeq RNA sample prep kit (Illumina). To do so, 300–1000 ng of total mRNA from mature seeds was used according to the manufacturer’s instructions. Sequencing was then performed on an Illumina HiSeq 1500, as a 2 × 101 paired-end run.

### Read processing and *de novo*transcriptome assembly

We used a high-speed desktop computer for all data analysis. Specifically, the hardware was a Dell Precision T1650 desktop computer with Intel Xeon E3-1270 (Quad Core), 32GB DDR3 1600MHz RAM, and 2TB Hard Drive. Data was stored locally on a suite of network attached hard drives, specifically four Western Digital Caviar Red NAS WD30EFRX, 3TB capacity each with SATA 6Gb/s interface, 3.5” form factor, IntelliPower RPM, and 64MB cache enclosed in a QNAP TS-412 1.2GHz 4 Bay enclosure.

The quality of the raw sequences was visually inspected using the open-source software FastQC (http://www.bioinformatics.babraham.ac.uk/projects/fastqc/). The FASTX toolkit (http://hannonlab.cshl.edu/fastx_toolkit/) was used to remove poor quality reads. *De novo* transcriptome assembly was performed with CLC Genomic Workbench 6.5.1. Two assembly methods were applied (Figure 2).

In the automatic word size, non-paired method, raw forward and reverse sequences were uploaded to CLC software indicating they were paired reads. CLC sorted these reads and stored them together in a single file. Reads in this single file were trimmed using the FASTQ quality trimmer tool in the FASTX toolkit to obtain a quality threshold of the read ends of 30 by maintaining the minimum length after trimming at 50. FASTQ quality filter tool removed poor quality reads to maintain a quality threshold at 30 and minimum percentage of bases that match the quality threshold 30 as 90. Then the reads were collapsed using the FASTX collapser tool to remove identical duplicates and uploaded to CLC Genomics as a FASTA file. This was assembled using the CLC *de novo* assembly under default settings (Graph parameters : Automatic word size - yes, Automatic bubble size - yes. Mapping options: Map reads back to contigs; Mismatch cost 2, Insertion cost 3, Deletion cost 3, Length fraction 0.8, Similarity fraction 0.8) except that the minimum contig length was set to 100 (default is 200). The word size and the bubble size set by the program in this case were 23 and 50 respectively for all datasets.

For the paired method, raw forward and reverse sequences were trimmed and filtered using the FASTX toolkit maintaining the same parameters as above. Filtered forward and reverse reads were uploaded into CLC Genomics indicating that they are paired reads. Different word sizes (*i.e*. 20, 35, 40, 45, 50, 60, 62, 63, and 64) as well as the automatic word size set by the program were tried. CLC default settings were used except the following changes. Minimum contig length: 300, Auto detect paired distances: yes, Perform scaffolding: yes.

Each assembly was searched for the presence of *PawS1* and *PawL1* genes using the tBLASTn procedure using the 151-residue sunflower PawS1 protein sequence as the query.

### Selecting core gene for quality assessment

Sunflower seed *de novo* transcriptome was assembled with the word size 60, paired method. Then reads were mapped back to contigs maintaining the parameters as below. Mismatch cost: 2, Insertion cost: 3, Deletion cost: 3, Length fraction: 0.8, Similarity fraction: 0.8, and Update contigs: Yes. Contigs were ordered based on their average coverage values. Six main subsets of contigs were selected to include contigs with average coverage value above 100,000, in the range of 10,000, 1,000, 500 (*PawS1* is in this range), 100, and contigs with average coverage values less than 100. These subsets of contigs were extracted and searched for their representative transcripts using the BLAST at NCBI tool. BLAST outputs for each subset were carefully examined and six core transcripts were selected to assess the quality of transcriptomes (Table 2). Care was taken to include transcripts coding for common plant proteins and mRNA that are expressed especially in seeds, also long and short transcripts. Dozens of Asteraceae *de novo* seed transcriptome assemblies were searched for these core genes and they appear to be conserved among all species tested.

Each transcriptome was queried for these core genes using the tBLASTn procedure. If several closely related transcripts were observed in the output the one with the best matching score was selected as the target gene. Coverage of identified core transcript was calculated as a percentage relatively to the length of the query protein. To evaluate the abundance of selected transcripts in each species at the mRNA level, trimmed and filtered raw reads were mapped to the ORF/partial ORF using the map reads to reference tool in CLC. Mapping parameters were maintained as Mismatch cost: 2, Insertion cost: 3, Deletion cost: 3, Length fraction: 0.8, Similarity fraction: 0.8.

### Peptide synthesis, peptide extract and LC-MS confirmation

Synthetic peptides used as standards in LC-MS were produced by Wuxi Nordisk Biotech Co., Ltd, China. Peptides were extracted from mature *Z. haageana* seeds as described [17]. Peptides were resuspended in 5% acetonitrile (v/v), 0.1% formic acid (v/v) and loaded onto a C18 high capacity nano LC chip trapping column (160 nL) (Agilent) in 95% Buffer A (0.1% formic acid) and 5% Buffer B (0.1% formic acid in acetonitrile) using a 1200 series capillary pump (Agilent). After loading samples, the trapping column was switched in-line with a 1200 series nano pump (Agilent) and a C18 analytical column (75 mm × 150 mm) and then using a gradient, 5% B to 60% B in 15 min, peptides were eluted into a 6510 Series QTOF mass spectrometer (Agilent). The QTOF was operated in MS-mode only. MS data were collected at eight spectra per second.

### Cloning *Z. haageana PawS1*and *PawL1*genes

Genomic DNA was extracted from mature *Z. haageana* seeds using the DNeasy Plant Mini Kit (Qiagen). Full length ORFs of *Z. haageana PawS1a* and *PawS1b* were specifically amplified using primers AJ58 (5′ CATC GAT CCT AGA AGA CAA TGG CAG TT 3′) and AJ59 (5′ GTG CGT AAG TGC GTA CAT TAA CCT 3′) and AJ60 (5′ GAT CCG ACT ACG ATG GCA GTT AAA G 3′) and AJ61 (5′ GAG TTA AAC ACA GAC CAC ACG 3′) respectively using *PfuUltra* high fidelity DNA polymerase (Agilent Technologies). In addition, the full length *PawL1a* was amplified with above genomic DNA with primers AJ134 (5′ TCC ACA ATG ACG AAA CTC ACA CTC 3′) and AJ56 (5′ ATC TTT ATT ACA AAT ACA TAC ATA GGC 3′) using *Taq*-DNA polymerase. Amplified fragments were cloned into pGEM-Teasy vector (Promega). Three independent clones were sequenced for each transcript to determine if there were errors introduced by *Taq* DNA polymerase.

*ZhPawS1d* was assembled only partially in two assemblies with different word sizes, but they were overlapping. The full ORF could be reconstructed by combining these overlapping partial sequences. Based on that reconstructed full length ORF, primers were designed to amplify *ZhPawS1d* gene. PCR with *Z. haageana* seed genomic DNA and primers AJ65 (5′ ATG GCA GTT AAA GTT GCA CTT G 3′) and AJ66 (5′ CAA GCC TAA TAG CTT GAG ACA G 3′) produced a broadly-migrating band on an agarose gel indicating multiple products ranging in size had been amplified. The products of this PCR reaction were cloned into pGEM-Teasy vector (Promega) and 41 independent clones were sequenced. Among these 41 clones *ZhPawS1a*, *ZhPawS1b*, *ZhPawS1d*, *ZhPawS1e*, and *ZhPawS1g* were present.

### Accession codes

*Zinnia haageana* genes have been deposited in GenBank under the accession codes [GenBank: KM243336-KM243341]. The sequence data used to assemble the *Z. haageana* and *H. helianthodes* transcriptomes were deposited in the National Centre for Biotechnology Information under BioSample accession numbers [SRA: SAMN02933922 and SAMN02933923] respectively.
